# Global Geographical Disparities in Untreated Caries of Permanent Teeth

**DOI:** 10.31661/gmj.v13iSP1.3734

**Published:** 2024-12-08

**Authors:** Seyed Masoud Sajedi, Saba Moalemi, Arezou Khosravi, Mahshid Alimohammadi, Seyed Saman Khademi, Marzieh Rahimi Sarab, Gholamreza Mojarab, Alireza Feizi, Mohamad Saleh Parsaei

**Affiliations:** ^1^ Department of Oral and Maxillofacial Medicine, Faculty of Dentistry, Shahed University, Tehran, Iran; ^2^ Faculty of Dentistry, Shiraz University of Medical Science, Shiraz, Iran; ^3^ Private Practice in Restorative Dentistry, Tehran

**Keywords:** Global Disparities, Untreated Dental Caries, Socioeconomic Factors, Oral Health Inequities, Preventive Dental Care

## Abstract

Background: Untreated dental caries in permanent teeth is a widespread public health concern. These disparities arise from complex interactions between socioeconomic, healthcare, and dietary factors, despite advancements in dental care and prevention strategies. This study aims to analyze global geographical disparities in untreated dental caries of permanent teeth and identify key systemic factors influencing its prevalence, such as dental workforce density, affordability of fluoride toothpaste, sugar consumption, and healthcare expenditure. Materials and Methods: An ecological study design was utilized, incorporating country-level data from the World Health Organization and related databases. Descriptive analyses, chi-square tests, and multiple linear regression models were employed to explore associations between untreated caries prevalence and independent variables. Sensitivity analyses addressed data outliers and regional variability. Results: The prevalence of untreated caries ranged from 22.8% to 49.5%, with a mean of 32.4% ± 4.8%. Dental workforce density was a statistically significant predictor (P = 0.014), with higher density associated with increased caries prevalence, suggesting underlying systemic inefficiencies. Other factors, such as sugar consumption and affordability of fluoride toothpaste, showed weak or nonsignificant associations. Sensitivity analyses confirmed the robustness of these findings, highlighting notable regional disparities and indirect influences on caries outcomes. Conclusion: Geographical disparities in untreated caries prevalence are driven by systemic factors, with evidence pointing to inefficiencies in the distribution and accessibility of oral healthcare resources. These findings emphasize the need for targeted public health policies that address regional inequities, improve preventive care access, and account for broader socioeconomic determinants.

## Introduction

Dental caries remains one of the most prevalent non-communicable diseases globally, affecting individuals across all age groups and socioeconomic strata [1]. Despite being largely preventable, untreated caries of permanent teeth is a significant public health concern, particularly in low- and middle-income countries [2].

The Global Burden of Disease (GBD) study underscored the pervasive impact of oral health disparities, estimating substantial years lived with disability attributable to untreated caries [3].

This silent epidemic, however, is not distributed equally across the globe; rather, it reveals stark geographical and socioeconomic inequities that underscore the need for targeted policy and intervention [4].

The consequences of untreated caries in permanent teeth extend beyond oral health, influencing systemic health, quality of life, and economic productivity [5].

Caries not only lead to pain, infection, and tooth loss but also contribute to absenteeism from school and work, reduced social interactions, and a significant economic burden on healthcare systems [6]. These impacts are magnified in resource-constrained settings where access to preventive and curative oral health services is limited, and individuals must often bear the costs of care out-of-pocket [7] Such disparities raise critical questions about the underlying determinants of untreated caries and the systemic barriers that perpetuate this health inequity [8].

Geographical disparities in untreated caries are shaped by complex interplays of social, economic, and environmental factors [9]. Socioeconomic status, dietary patterns, access to fluoride, oral hygiene practices, and availability of dental care infrastructure vary significantly between and within countries, driving differences in caries prevalence and treatment rates [10]. High-income countries have made significant strides in reducing caries through the widespread use of fluoridated products, organized preventive programs, and better access to dental care [11].

In contrast, many low- and middle-income countries continue to experience a rising burden of caries due to increased consumption of sugary diets, poor oral hygiene, and insufficient access to fluoride [12][13]. This bifurcation highlights the inequities in oral health outcomes that are closely tied to broader global development challenges [14].

Furthermore, ecological studies that examine untreated caries through a geographical lens provide valuable insights into patterns and determinants that may not be apparent at the individual level [15]. By aggregating data across regions or countries, these studies help illuminate systemic disparities and inform macro-level interventions [16]. For instance, regional policies on fluoride use, public health expenditures, and cultural attitudes toward dental care can have a profound influence on the prevalence of untreated caries [17]. However, current literature on this subject often lacks comprehensive global analyses that account for regional variations, limiting the ability to formulate universally applicable solutions [18].

This study examines the geographical disparities in untreated caries of permanent teeth, focusing on factors such as the affordability of fluoride toothpaste, availability of dental professionals, healthcare expenditure, and sugar consumption. By analyzing data at the global level, we aim to identify key determinants and contextualize disparities in untreated caries prevalence worldwide.

## Methods and Materials

Study Design

This ecological study aimed to investigate global geographical disparities in untreated caries of permanent teeth and their association with socioeconomic, healthcare, and dietary factors. Country-level data were collected and analyzed to identify potential determinants of untreated caries prevalence. The study utilized publicly available data obtained primarily from the World Health Organization (WHO) and other global databases.

Data Collection and Processing

Data for this study were collected from publicly available World Health Organization (WHO), Global Health Observatory (GHO), and related publicly accessible global health datasets. Available here: https://www.who.int/data/gho All variables were collected for the most recent year available, typically between 2019 and 2023, ensuring.

The primary outcome variable, the prevalence of untreated caries in permanent teeth, was extracted from WHO’s oral health surveillance reports. Independent variables, reflecting socioeconomic, healthcare, and behavioral factors, were also derived from WHO data and included:

• Affordability of Fluoride Toothpaste: Number of labor days required to buy an annual supply per person.

• Dental Workforce Density: Number of dentists per 10,000 population.

• Sugar Consumption: Per capita availability of sugar (grams/day).

• Dental Healthcare Expenditure: Per capita expenditure on dental healthcare (US$).

• Oral Health Screening: Proportion of the population covered by routine oral health screenings.

• Preventive Dental Care: Availability and routine access to preventive oral healthcare services.

The collected data underwent a thorough cleaning and processing protocol. Missing data for individual variables were addressed by applying imputation techniques where feasible, or by excluding countries with substantial data gaps for critical variables. To ensure consistency across data from various sources, variables with differing measurement units were converted to standardized formats (e.g., per capita measures or z-scores).

Data were further verified by cross-referencing multiple global health reports and statistical publications. The analysis included countries and regions with complete datasets for the variables of interest. Countries or regions with incomplete information for the primary outcome variable were excluded from the analysis. The final dataset included all countries with complete data for both the prevalence of untreated caries and at least 80% of the independent variables, ensuring a robust analytical sample.

Statistical Analysis

Descriptive statistics, including mean values and standard deviations, were calculated for all continuous variables, while categorical variables were summarized as frequencies and percentages. These statistics provided an overview of the characteristics of countries included in the analysis. Additionally, a correlation matrix was constructed to evaluate the relationships between independent variables and the prevalence of untreated caries.

Using these descriptive results, significant associations were identified between the outcome variable and predictors. Pearson chi-square tests were used to assess the associations between categorical independent variables and the prevalence of untreated caries. Independent variables that showed significant associations (P < 0.05) in the chi-square tests were included in multivariable regression models.

Multiple linear regression models were then performed to identify the factors associated with untreated caries prevalence. After initial model construction, multicollinearity among independent variables was assessed using Variance Inflation Factor (VIF), and highly collinear variables (VIF>5) were excluded from the final models. Sensitivity analyses were conducted to ensure the robustness of the results, with outlier countries excluded and results stratified by geographic region

All statistical analyses were conducted in Python (version 3.11) with packages including Pandas for data manipulation and Stats models for regression analysis. For visualization, we used Seaborn and Matplotlib and © 2024 Datawrapper is developed by Datawrapper GmbH.

Ethical Considerations

This study used publicly available, aggregated data and did not involve human participants. Therefore, ethical approval was not required. Data sources were appropriately cited to ensure transparency.

## Results

**Figure-1:**
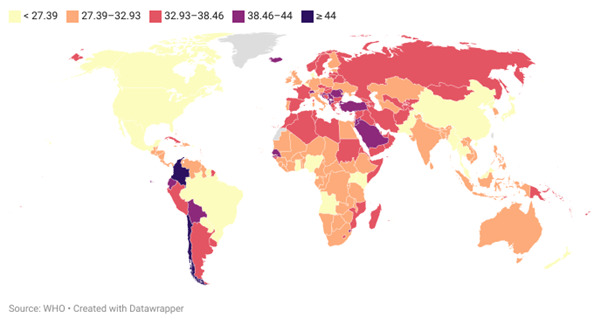
Figure 1. Global distribution of untreated dental caries prevalence.

**Figure-2:**
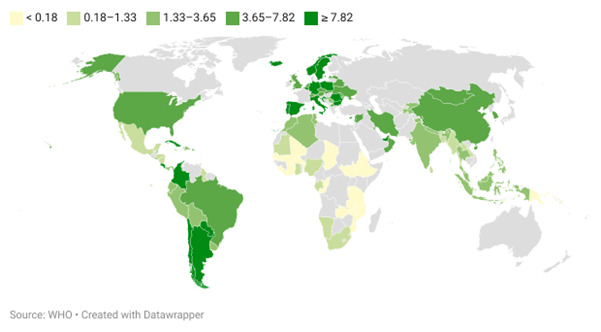
Figure 2. Global distribution of dental workforce density per 10,000 population.

**Figure-3:**
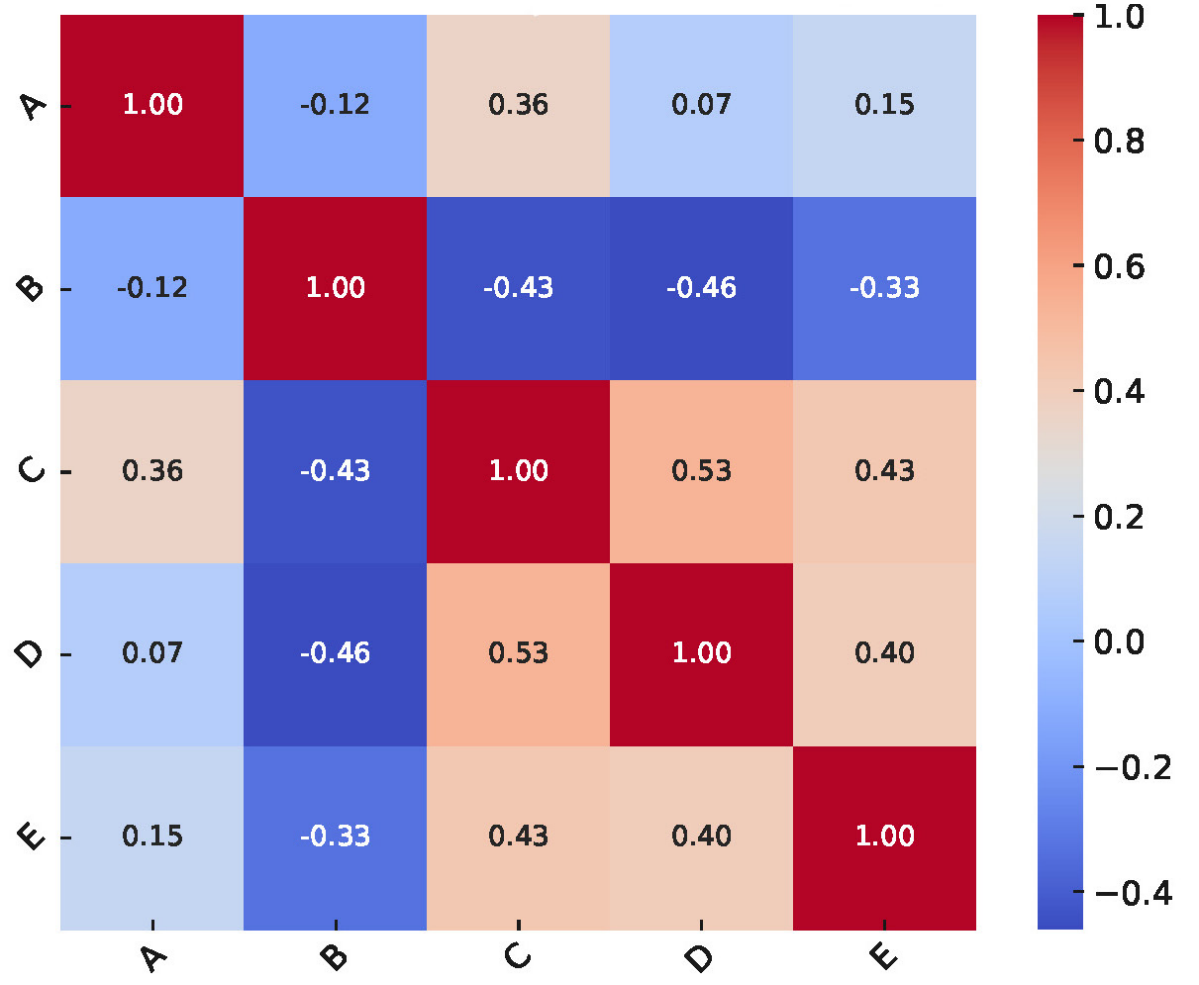
Figure 3. Correlation Heatmap of Variables A: Prevalence of untreated caries of permanent teeth (%) B: Number of labour days needed to buy annual supply of fluoride toothpaste per person C: Dentists (per 10,000) D: Per capita availability of refined sugar (g/day) E: Per capita expenditure on dental healthcare (US$)

**Figure-4:**
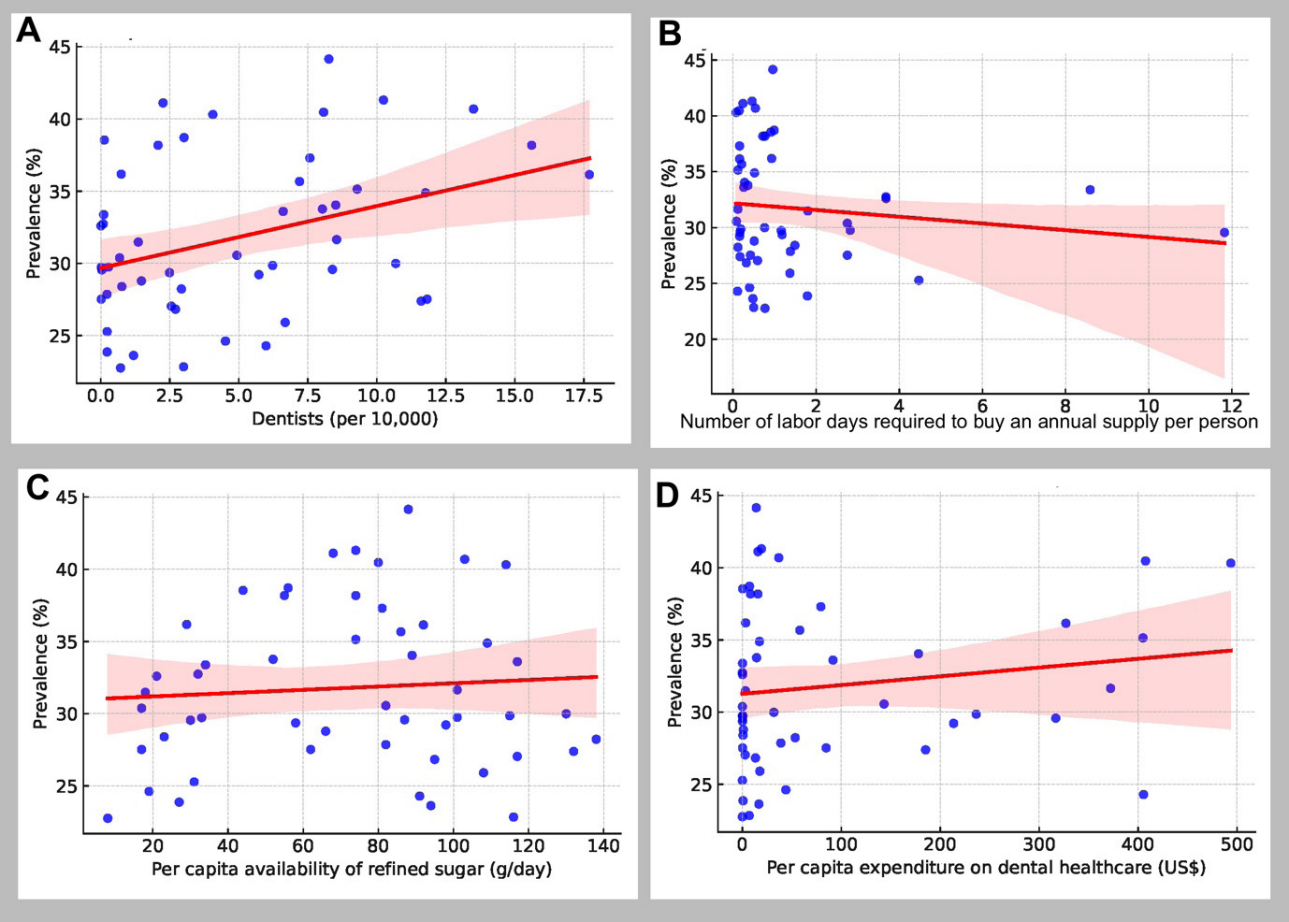
Figure 4. Scatter plots with regression lines, depicting the relationships between individual predictors and the prevalence of untreated caries in permanent teeth (%). A: Dentists (per 10,000) B: Number of labor days required to buy an annual supply per person. C: Per capita availability of refined sugar (g/day) D: Per capita expenditure on dental healthcare (US$)

**Figure-5:**
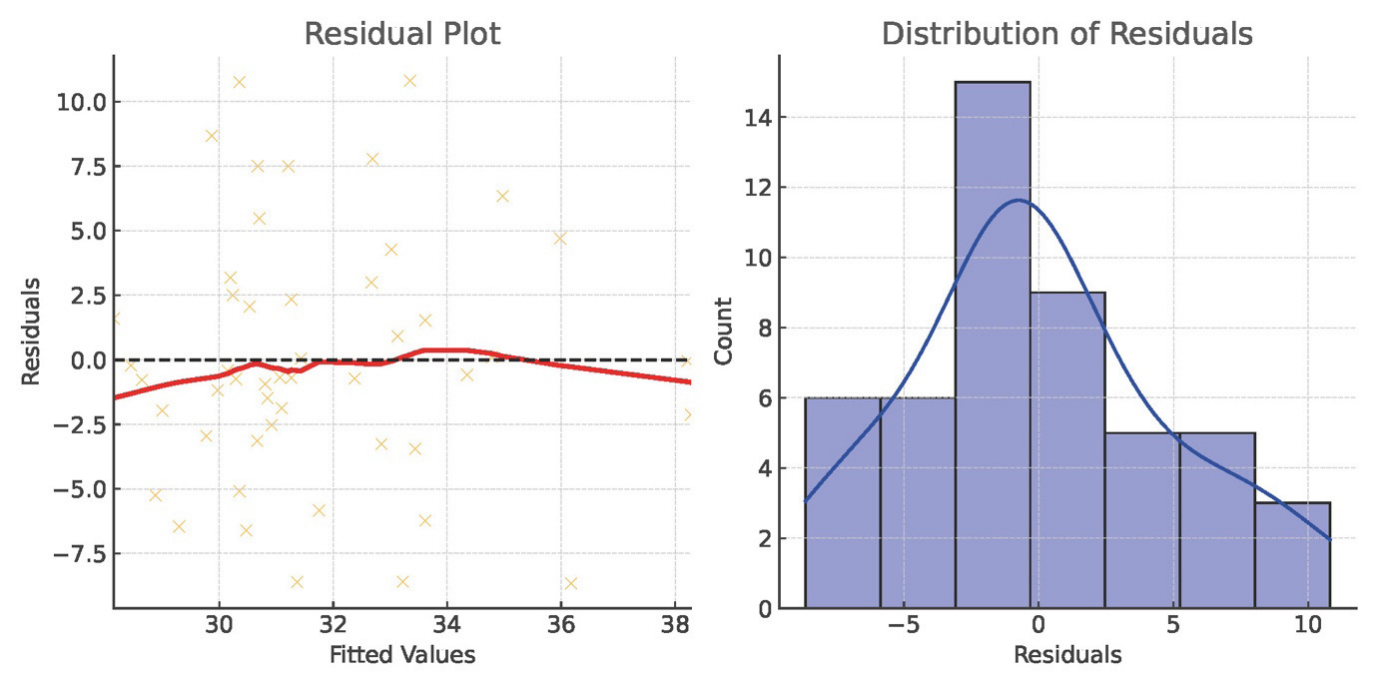
Figure 5. 5. Distribution of Residuals

**Table:** Table1. Descriptive Statistics of Variables

**Variables**	**Count**	**Mean**	**SD**	**Min**	**25%**	**50%**	**75%**	**Max**
prevalence of dental caries (%)	111	32.41	4.81	22.76	29.24	31.7	35.65	49.53
Dental Workforce Density	110	4	4.51	0.007	0.28	2.26	6.60	17.7
Sugar Consumption (g/day)	103	70.06	35.24	7	35.5	72	96.5	141
Affordability of Fluoride Toothpaste	52	1.26	2.10	0.07	0.18	0.51	1.22	11.82
Dental Healthcare Expenditure (US$)	111	50.14	102.70	0.0006	0.49	7.28	34.45	493.7

**Table:** Table2. Details the results of multiple linear regression analysis.

Variable	Coefficient	Standard Error	t-Statistic	P-Value
Affordability of Fluoride Toothpaste	0.021	0.416	0.051	0.96
Dental Workforce Density	0.523	0.205	2.559	0.014
Sugar Consumption (g/day)	-0.025	0.026	-0.969	0.338
Dental Healthcare Expenditure (US$)	0.001	0.006	0.187	0.853
Oral Health Screening	-	-	-	0.86
Preventive Dental Care	-	-	-	0.88

**Table:** Table3. Associations Between Categorical Predictors and The Prevalence of Untreated Dental Caries

Variable	Chi-Square	P-value	Degrees of Freedom
Availability and routine access to preventive oral healthcare services.	0.70	0.40	1
Oral health screening for early detection of oral diseases	0.36	0.83	2

The mean ± SD prevalence of untreated caries across the dataset was 32.4% ± 4.8%, with notable disparities across geographic regions and socioeconomic indicators. The highest prevalence recorded was 49.5%, while the lowest was 22.8%. Figure-1 illustrates the global variation in untreated dental caries prevalence, highlighting disparities across regions.

The average dental workforce density was 4.92 ± 4.62 dentists per 10,000 population, with significant regional differences in distribution, as shown in Figure-2.

Detailed descriptive statistics for other variables are provided in Table-1. Among binary variables, 91 (82%) countries reported the availability of routine preventive oral health care services, while only 28 (25%) countries implemented oral health screening programs aimed at early disease detection.

A multiple linear regression analysis identified dental workforce density as a significant predictor of untreated caries prevalence (P = 0.014). Counterintuitively, higher dental workforce density was associated with a greater prevalence of untreated caries; a one-unit increase in dental workforce density corresponded to a 0.52% increase in untreated caries prevalence. This finding suggests the influence of indirect or confounding factors, such as uneven distribution of dental services or reporting biases. Other predictors, including the number of labor days required to purchase fluoride toothpaste, per capita sugar availability, and expenditure on dental healthcare, were not statistically significant (P > 0.05). Table-2 provides a detailed summary of the regression results, while Table-3 outlines associations between categorical predictors and dental caries prevalence.

A correlation heatmap (Figure-3) revealed a moderate positive correlation (r = 0.51) between dental workforce density and per capita sugar availability, suggesting potential indirect pathways influencing caries prevalence. Scatter plot analyses (Figure-4) illustrated weak trends between individual predictors and untreated caries prevalence. Notably, per capita sugar availability demonstrated a slight inverse association with untreated caries prevalence, though this relationship was not statistically significant. Similarly, the number of labor days for fluoride toothpaste showed negligible association with caries prevalence.

Sensitivity analyses were performed to evaluate the robustness of the regression model. After removing outliers identified through Cook’s Distance, the adjusted R² improved to 0.186, and dental workforce density remained a significant predictor (P = 0.014). Multicollinearity diagnostics indicated no severe issues, with all Variance Inflation Factor (VIF) values below the threshold of 5 (Figure-5), ensuring reliable regression coefficients.

Stratified regression analyses by geographic region confirmed the significance of dental workforce density in influencing untreated caries prevalence in the "Other" category (P = 0.017).

However, results for other regional categories did not yield statistically significant associations, highlighting the need for further investigation into region-specific factors. The findings highlight how socioeconomic and healthcare factors interact to shape untreated caries prevalence globally, emphasizing systemic inequities that require targeted interventions.

## Discussion

This study highlights significant global disparities in the prevalence of untreated caries in permanent teeth, driven by geographic and systemic inequities. The findings provide critical insights into how socioeconomic and healthcare factors influence oral health outcomes, offering valuable implications for both theoretical models and public health policies.

Our analysis reveals a wide range of untreated caries prevalence globally (mean: 32.4%; range: 22.8%-49.5%), underscoring stark inequities in access to preventive resources and dental care. These disparities align with previous research emphasizing oral health as a marker of broader systemic inequity [3]. The observed variability suggests that untreated caries is not solely a function of individual behaviors but rather reflects deeper structural determinants that require targeted interventions [2].

The counterintuitive positive association between dental workforce density and untreated caries prevalence likely reflects systemic inefficiencies, such as the concentration of dental professionals in urban centers while rural areas remain underserved. Also, regions with higher dental workforce density may prioritize curative care over preventive measures, or the relationship may be influenced by enhanced disease reporting accuracy in these regions. Further research, incorporating spatial distribution data and service type categorization, is necessary to elucidate these dynamics [19]. Alternatively, regions with higher dental workforce density may focus predominantly on restorative or emergency care rather than preventive services [20]. The association could also reflect better documentation and reporting in these regions rather than a true increase in disease burden [21]. Future research should explore these mechanisms further, particularly by examining the spatial distribution patterns of dental professionals and the nature of services provided [22].

Variables such as fluoride toothpaste affordability, sugar consumption, and healthcare expenditure showed weak or non-significant associations with untreated caries prevalence. However, these results may oversimplify complex dynamics. For example, fluoride toothpaste affordability does not guarantee usage without adequate public health education, while sugar consumption effects may be mitigated by regional dietary patterns, access to fluoridated water, or oral hygiene practices. Future studies should explore these variables within a broader behavioral and systemic framework to uncover potential interactions and confounders. These results point to the multifactorial etiology of dental caries, where cultural attitudes, behavioral practices, and systemic healthcare barriers may overshadow the direct impact of these predictors. For instance, the affordability of fluoride toothpaste may not translate into widespread use without adequate public health education or awareness campaigns [23]. Similarly, sugar consumption, while a well-established risk factor for caries, may have its effects mitigated by factors such as dietary patterns, water fluoridation, or oral hygiene practices [24][25]. These nuances warrant further investigation to uncover potential confounders and regional variations in these relationships [26].

The ecological design of this study allowed for the examination of systemic and regional patterns, but it also introduced inherent limitations. Aggregated data, while useful for identifying macro-level trends, cannot capture individual-level behaviors or outcomes, raising the possibility of ecological fallacy [27]. For example, while regions with higher dental workforce density reported greater caries prevalence, this association may not hold for individuals within those regions. Additionally, the cross-sectional nature of the data precludes causal inferences, emphasizing the need for longitudinal studies to track changes in caries prevalence over time and establish temporal relationships among variables [28].

Excluding countries with incomplete data may introduce selection bias, disproportionately representing regions with advanced healthcare infrastructure and reliable data collection. This could underestimate the true burden of untreated caries in low-resource settings. Using imputation techniques or alternative methods to handle missing data could mitigate this bias and provide a more representative analysis of global disparities. This limitation highlights the importance of improving global oral health surveillance and ensuring the representation of low-resource settings in future research.

Despite these limitations, the study advances understanding of oral health disparities by emphasizing systemic inequities and the need for integrated, context-specific interventions. The findings suggest that addressing untreated caries requires a multi-pronged approach, including equitable distribution of dental resources, expansion of preventive care, and culturally tailored public health education programs. Strategies such as mobile dental clinics, tele-dentistry initiatives, and incentives for dentists to serve underserved areas could help bridge urban-rural gaps in access to care [29]. Integrating oral health into broader public health frameworks, such as routine screenings in primary care, could also facilitate early detection and prevention, particularly among vulnerable populations [30].

Future research should prioritize longitudinal studies to uncover causal pathways, region-specific analyses to tailor interventions, and evaluation of public health programs to identify cost-effective strategies. By addressing the structural and systemic determinants of oral health inequities, we can make meaningful progress in reducing the global burden of untreated caries and improving population health outcomes.

Limitations

This study has several limitations that should be acknowledged. First, the ecological design relies on aggregated country-level data rather than individual-level data, introducing the potential for ecological fallacy. Associations observed at the national level may not reflect relationships at the individual level, limiting the precision of inferences. Also, the cross-sectional design limits causal inferences, as temporal relationships between predictors and untreated caries prevalence cannot be established. To overcome this, future research should adopt longitudinal approaches that track changes in prevalence and predictors over time, enabling the identification of causal pathways and feedback loops. Temporal relationships and potential feedback loops, such as how untreated caries might influence healthcare expenditures or service accessibility, cannot be fully explored.

Data availability posed another significant limitation, as countries with incomplete data were excluded from the analysis. This exclusion likely introduced selection bias, disproportionately representing nations with robust healthcare systems and underestimating the burden of untreated caries in low-resource settings. Furthermore, key variables such as dental workforce density and sugar consumption, while useful at the macro level, oversimplify complex dynamics, such as the uneven distribution of dental professionals and the interplay of dietary habits with preventive practices. Lastly, reliance on secondary data from global databases may have affected the reliability and comparability of results due to inconsistencies in data collection and reporting methods across countries. Despite these limitations, the study provides critical insights into global disparities in untreated caries and underscores the need for targeted interventions.

## Conclusion

This study highlights significant disparities in untreated caries prevalence, driven by systemic and geographic inequities. Addressing these disparities requires equitable resource distribution, enhanced preventive care, and culturally tailored oral health education. By prioritizing interventions that target structural determinants, public health systems can reduce the global burden of untreated caries and improve oral health outcomes. Future research should focus on exploring these indirect pathways and region-specific determinants to further inform effective strategies for reducing the global burden of untreated caries.

## Conflict of Interest

Dr. Seyed Massoud Sajedi serves as a guest editor for Galen Medical Journal and is also an author of the submitted paper. To ensure the integrity and impartiality of the editorial process, He has recused himself from any editorial duties or decisions related to this submission. An independent editor has been assigned to oversee the peer review and decision-making process for this manuscript, by the journal’s conflict of interest policy. Expect this issue, He and other authors have declared that they have no conflicts of interest to disclose.
